# Study on Pitting Corrosion Simulation of Steel Plates Based on Cellular Automaton-Finite Element Coupling

**DOI:** 10.3390/ma19051001

**Published:** 2026-03-05

**Authors:** Shizhong Liu, Wei Zhang

**Affiliations:** 1 Capital Construction Office of Xiangtan University, Xiangtan University, Xiangtan 411105, China; liuk9896@126.com; 2 School of Mechanical Engineering and Mechanics, Xiangtan University, Xiangtan 411105, China

**Keywords:** localized pitting corrosion, chloride ion cyclic corrosion (CICC) testing, cellular automata-finite element coupling, electro-thermo-mechanical-chemical (ETMC) multi-field coupling

## Abstract

Pitting corrosion is a prevalent and highly detrimental form of localized corrosion, which can severely compromise the local load-bearing capacity of metallic materials and, in extreme cases, trigger structural failure. In response to the pronounced susceptibility of Q235 galvanized steel plates to localized pitting under the extreme service conditions of the South China Sea—characterized by high temperature, high salinity, high humidity, and coupled chemical corrosive effects—this study conducts a systematic investigation combining experimental characterization and numerical simulation. First, a novel accelerated pitting corrosion apparatus was designed and developed, and chloride ion cyclic corrosion (CICC) tests were performed on Q235 galvanized steel plates. The morphology and temporal evolution of pitting damage were comprehensively characterized. Subsequently, based on a coupled Cellular Automata (CA) and Finite Element Analysis (FEA) framework, a corrosion evolution model termed CAFE (Cellular Automata-Finite Element) was established. This model elucidates the initiation, growth, and corrosion product evolution of pitting pits under varying temperature and salinity conditions and further quantifies the spatial distributions of stress and temperature fields in the vicinity of pitting sites. Finally, experimental results were employed to validate the rationality and effectiveness of the proposed electro-thermo-mechanical-chemical (ETMC) multi-field coupling model. The results demonstrate that temperature and salinity are the dominant environmental parameters governing the evolution of localized pitting corrosion rates. A strong agreement between numerical predictions and experimental observations is achieved in both qualitative trends and quantitative metrics. Notably, the model reveals that under elevated current-driving conditions, localized plastic deformation plays a critical role in promoting pit propagation and accelerating the pitting corrosion process.

## 1. Introduction

Steel plates subjected to thermal treatment and pre-tensioning processes can exhibit markedly enhanced mechanical performance and corrosion resistance, which has led to their widespread application in coastal building structures [1], shipbuilding, and marine engineering equipment [2,3,4,5,6]. However, during long-term service in complex marine environments characterized by high salinity, high humidity [7], and elevated temperatures [1,6], such structural components are continuously exposed not only to electrochemical corrosion but also to external mechanical loading and environmental fields, resulting in pronounced multi-field coupling effects [3,7]. Under these conditions, elucidating the evolution mechanisms of corrosion-induced damage in materials subjected to electro-thermo-mechanical-chemical (ETMC) coupling has emerged as a critical scientific challenge for structural safety assessment and service-life prediction.

Compared with corrosion processes governed by a single environmental factor, corrosion behavior under ETMC multi-field coupling exhibits significantly enhanced nonlinearity, strong time dependence, and pronounced local instability. Existing studies indicate that corrosion damage accumulates progressively over service time, while current-induced Joule heating can trigger localized thermally induced plastic deformation, thereby accelerating corrosion development. Under high-current-density conditions, severe stress concentration further promotes rapid pit propagation and may even lead to perforation failure. Nevertheless, owing to the high cost, long duration, and inherent difficulty in capturing the full temporal evolution of corrosion processes in real time, experimental approaches alone remain insufficient to comprehensively reveal the intrinsic mechanisms governing corrosion evolution under complex service conditions.

In recent years, advances in computational materials science and numerical simulation techniques have established computational modeling as an increasingly important complementary tool for investigating corrosion mechanisms. Among the available approaches, Cellular Automata (CA) models have attracted growing attention owing to their intrinsic ability to capture localized interactions, their adaptability to complex evolutionary processes, and their high scalability in multi-field coupling frameworks. These features endow CA models with distinct advantages in the visualization and simulation of corrosion morphology evolution, providing an effective modeling paradigm for corrosion problems characterized by pronounced randomness and multi-scale features.

At the mesoscale, CA models have been widely employed to describe the spatiotemporal evolution of corrosion morphologies [8,9,10,11,12,13,14,15,16,17,18]. For instance, Chen et al. [19] reconstructed pitting morphologies on Q235 steel surfaces using a CA-based approach, achieving good agreement with experimental observations; however, their analysis was restricted to two-dimensional representations. Wang et al. [20] investigated the evolution of corrosion current under varying environmental parameters, yet the study lacked corresponding experimental validation. Guo et al. [21] further extended CA modeling to three-dimensional domains and incorporated temperature and concentration effects to examine pit growth behavior, but mechanical responses and finite element analyses were not considered. While these studies provide valuable theoretical foundations and computational tools for durability assessment of metallic structures, the corrosion-mechanics interaction under multi-field coupling conditions remains insufficiently characterized.

To address these limitations, coupled CA-finite element (CAFE) modeling strategies have been developed in recent years [22,23,24]. By integrating stress and strain-based mechanical responses into the simulation of corrosion morphology evolution, CAFE models offer a promising framework for investigating localized corrosion behavior under external loading. For example, Fatoba et al. [22] employed a CA-FE coupling approach to systematically analyze the effects of applied loads and electrochemical parameters on pit depth, depth-to-width ratio, and morphological evolution and further discussed the mechanistic roles of stress field distributions, metal-ion hydrolysis, corrosion product accumulation, and oxidation processes in pit propagation. These studies collectively demonstrate that synergistic CA-finite element modeling can more faithfully capture the dynamic evolution of material corrosion under complex service environments. Nevertheless, most existing CAFE frameworks remain predominantly two-dimensional, and a systematic description of three-dimensional corrosion behavior and its interaction with multi-field coupling effects is still lacking.

As a representative form of localized corrosion, pitting corrosion is closely associated with the localized breakdown of protective passive films on material surfaces and is particularly susceptible to chloride ion attack in marine environments. Although the underlying mechanisms of pitting corrosion have been elucidated to some extent, a systematic understanding of pitting evolution in commonly used engineering materials—such as carbon steel and galvanized steel—under extreme service conditions involving high current density, elevated temperature, and high salinity remains limited. Correspondingly, numerical modeling efforts addressing pitting corrosion under such conditions are still relatively underdeveloped. Existing modeling approaches include empirical models [25,26,27], electrochemical models, finite element models [1,25,28,29,30,31,32,33,34,35,36,37,38,39,40,41,42], statistical models [5,43], and three-dimensional damage peridynamic models [44]. However, owing to the pronounced nonlinearity and stochasticity inherent in corrosion processes, the applicability of these models under strongly coupled multi-field conditions requires further improvement.

Motivated by these limitations, this study focuses on the localized pitting mechanisms of Q235 galvanized steel plates under high-current conditions representative of the high-temperature, high-salinity, and high-humidity marine environment of the South China Sea. A novel pitting corrosion experimental apparatus is designed and developed in conjunction with chloride ion cyclic accelerated corrosion testing to obtain representative experimental data on pitting evolution. Building upon these observations, a three-dimensional CAFE corrosion modeling framework coupling cellular automata and finite element methods is proposed to simulate pit initiation, growth, and the evolution of corrosion products. The results demonstrate the accuracy and feasibility of the proposed approach in capturing corrosion behavior under electro-thermo-mechanical-chemical (ETMC) multi-field coupling, providing a robust theoretical basis and numerical tool for structural integrity assessment and service-life prediction under complex marine service conditions.

## 2. Experimental Section

In this study, a systematic experimental investigation was conducted on Q235 steel under controlled electrochemical laboratory conditions. Q235 steel is a typical carbon structural steel widely used in petroleum engineering and offshore platform structures. Its chemical composition is summarized in Table 1. The specimens employed in the experiments were galvanized Q235 steel plates with a diameter of 50 mm and a thickness of 5 mm.

**Figure 1:**
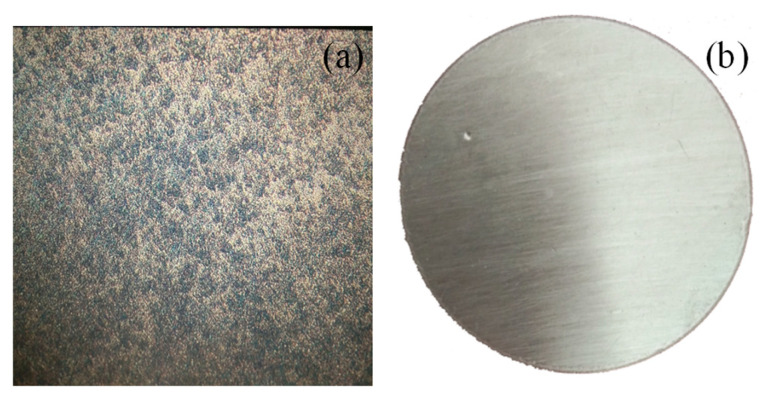
Micro-composition, specimens. (**a**) metallographic analysis; (**b**) specimens.

Mechanical property tests indicate that the steel exhibits a yield strength of 235 MPa at 0.2% strain, an ultimate tensile strength of 350 MPa, and an elongation at fracture of approximately 25%. Metallographic analysis reveals that the microstructure consists predominantly of ferrite and pearlite with a relatively uniform distribution, which is characteristic of typical low-carbon steel (see Figure 1).

**Figure 2:**
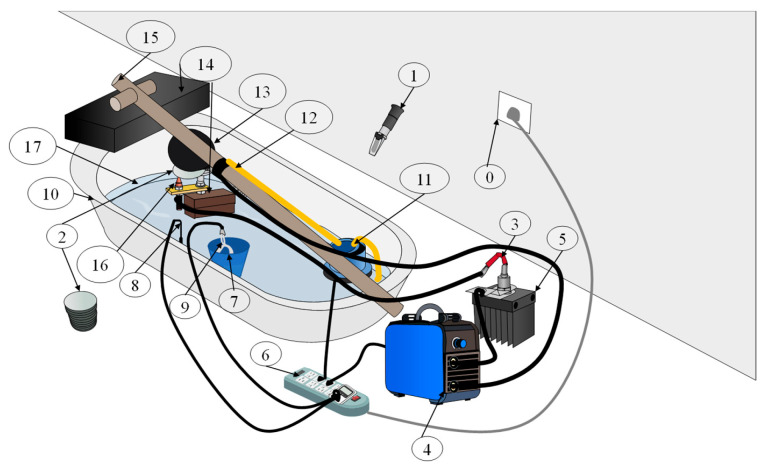
Layout of the experimental site.

Figure 2 illustrates the integrated experimental setup employed in this study. The apparatus accelerates the corrosion process in a controlled manner by enhancing electrochemical reactions on the steel surface through the application of an external current. The experimental system consists primarily of a power supply module, an electrolytic corrosion module, a temperature control unit, and a solution circulation system; the detailed configuration and component numbering are provided in the annotations of Figure 2.

During the experiments, a constant external current was applied in combination with a chloride ion cyclic mechanism, while the salinity and temperature of the solution were precisely controlled. This approach enables the accelerated simulation of corrosion processes in steel plates under representative marine conditions characteristic of the South China Sea.

The components labeled 0–17 correspond to:

0, power supply; 1, salinity meter; 2, galvanized steel plate (circular); 3, ZP300A diode; 4, welding power source; 5, heat sink; 6, power strip; 7, steel container; 8, temperature control sensor; 9, heating rod; 10, bath tank; 11, water pump; 12, water pipe; 13, steel spray nozzle; 14, spacer block; 15, support frame; 16, magnetic fixture; and 17, seawater.

**Figure 3:**
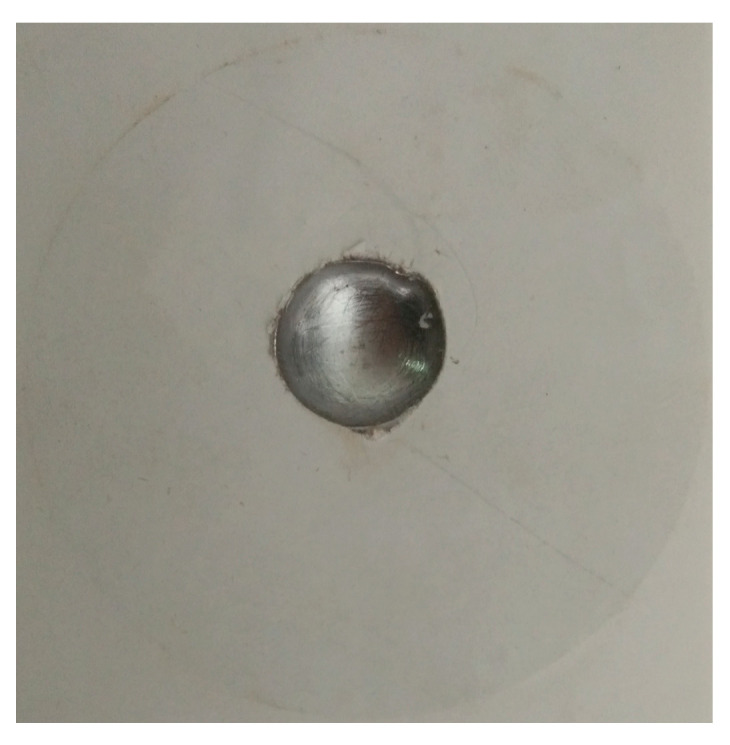
Localized corrosion pits.

In the localized pitting corrosion experiments, the galvanized coating was mechanically removed from the specimen surface prior to testing to eliminate its potential interference with the corrosion behavior. As shown in Figure 3, a single initial pitting site was pre-fabricated on the specimen surface using a twist drill. To spatially confine the corrosion region, a white adhesive masking film was applied to the surface outside the pit area, thereby ensuring good reproducibility and spatial controllability of the pitting process. Figure 3 also presents representative pit morphology evolution during the electrochemical corrosion process.

Experimental observations indicate that the cross-sectional profiles of the obtained pits generally exhibit a quasi-ellipsoidal geometry, with an approximately circular projection on the specimen surface. The sidewall profiles of the pits can be well described using a geometric model whose generatrix follows a second-order parabolic function (see Figure 3). Based on these experimental observations, a CA model calibrated with measured pit geometries was introduced in the subsequent numerical analysis. Experimental measurements obtained at different time scales were further employed to inversely determine and calibrate the geometric parameters of the pits. The geometric dimensions of the corrosion features were measured using a Mitutoyo Measuring Instruments (Shanghai) Co., Ltd., Shanghai, China with an accuracy of 0.01 mm and a high-precision electronic digital depth gauge (measurement range: 0–12.7 mm) with a resolution of 0.001 mm. These instruments ensured adequate dimensional accuracy and repeatability for quantifying pit width and depth during the corrosion evolution process.

Furthermore, as illustrated in Figure 3, pitting growth experiments were conducted under coupled electro-thermal conditions to systematically investigate the effects of solution salinity and ambient temperature on the evolution behavior of stabilized pits. Three NaCl solution concentrations, 3‰, 15‰, and 35‰, were selected, together with three ambient temperature levels of 10 °C, 28 °C, and 45 °C. These parameter combinations effectively cover the characteristic high-salinity, high-temperature, and high-humidity service conditions typical of the South China Sea.

The experiments were performed using a self-developed electrochemical-thermal coupling test system. By controlling the applied current density, solution temperature, and salinity, the influence of thermal effects and chloride ion concentration on pitting growth behavior was systematically analyzed. Key indicators characterizing the temporal evolution of the pitting process include the maximum pit depth, the pit diameter-to-depth ratio, and the overall pit morphology. The resulting experimental data provide a robust basis for parameter calibration and validation of the subsequent multi-field coupled numerical models.

## 3. Experimental Results and Discussion

### 3.1. Effect of Different Salinity Levels on Corrosion Pits

**Figure 4:**
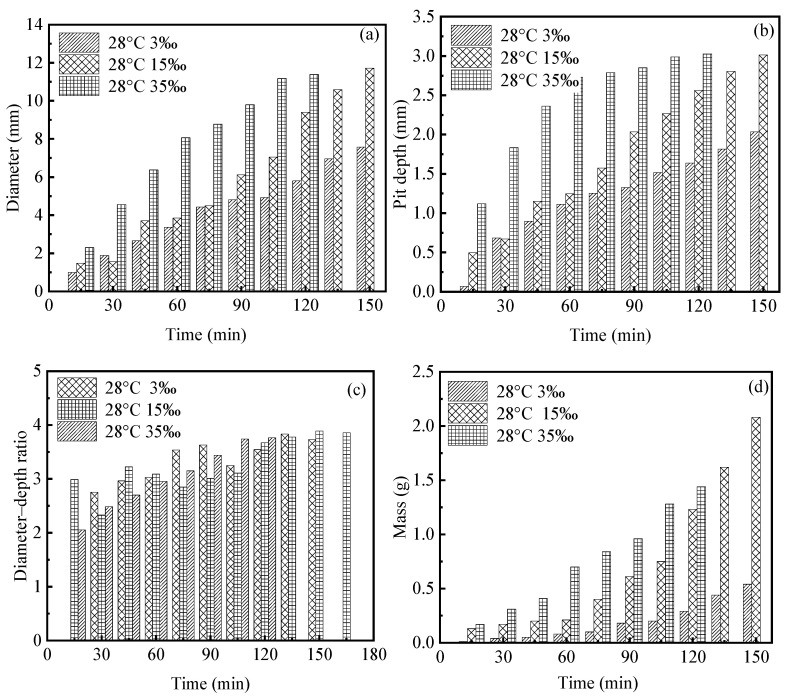
Effect of different NaCl solution concentrations on corrosion pits: (**a**) evolution of pit diameter over time at various salinities; (**b**) evolution of pit depth over time at various salinities; (**c**) evolution of pit diameter-to-depth ratio over time at various salinities; (**d**) evolution of corrosion mass over time at various salinities.

Figure 4a,b illustrate the effects of salinity on the temporal evolution of pit diameter and pit depth under a constant temperature of 28 °C. The results demonstrate that both pit diameter and depth increase markedly with increasing solution salinity and continue to grow throughout the corrosion process. Compared with low-salinity environments, pit growth under high-salinity conditions proceeds at a substantially faster rate, and the differences among various salinity levels become increasingly pronounced with prolonged exposure time. These observations indicate that salinity plays a dominant role in governing the evolution of pitting corrosion.

Figure 4c presents the evolution of the pit diameter-to-depth ratio as a function of time under different salinity conditions. It can be observed that, for all salinity levels, the diameter-to-depth ratio exhibits certain fluctuations during the early stages of corrosion, followed by a gradual stabilization and eventual convergence to an approximate constant value of 3.8. This behavior suggests that, under the present experimental conditions, although salinity significantly affects the growth rate of pitting corrosion, its influence on the overall geometric proportion of pits is relatively limited. As pitting progresses, the pit morphology tends to evolve toward a stable geometric similarity.

Furthermore, Figure 4d compares the time-dependent evolution of corrosion-induced mass loss under different salinity conditions. At the same temperature, the corrosion mass loss increases monotonically with time, and higher salinity results in more pronounced material loss, reflecting a more intense corrosion reaction. This trend is in good agreement with the observed evolution of pit size.

Taken together, these results indicate that increasing ionic concentration in the solution enhances the electrical conductivity of the electrolyte, thereby facilitating electrochemical reactions and accelerating metal dissolution. Meanwhile, thermally activated effects at elevated temperatures further intensify corrosion kinetics, ultimately leading to the formation of deeper and larger localized corrosion pits.

### 3.2. Effect of Different Temperatures on Corrosion Pits

**Figure 5:**
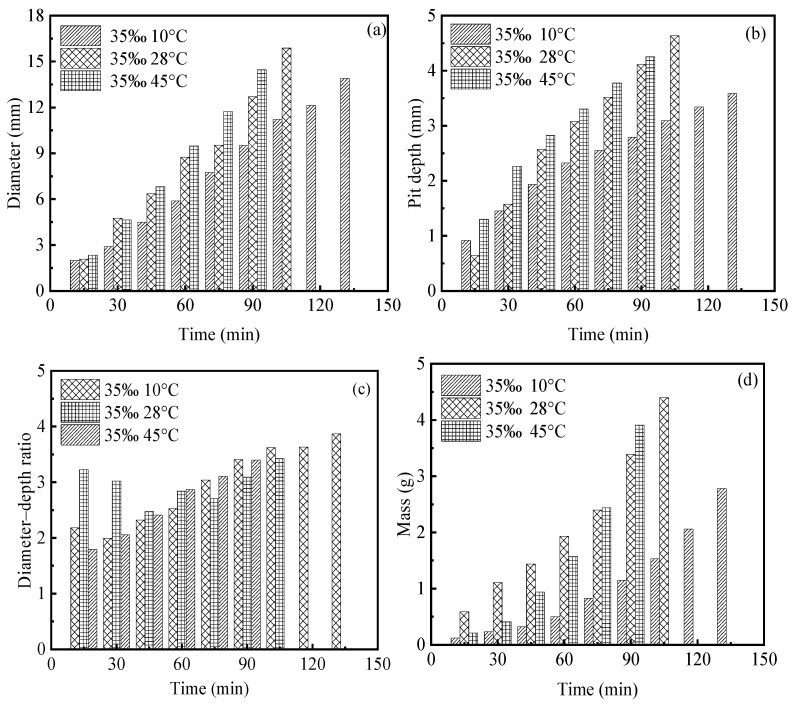
Effect of different temperatures on corrosion pits: (**a**) evolution of pit diameter over time at various temperatures; (**b**) evolution of pit depth over time at various temperatures; (**c**) evolution of pit diameter-to-depth ratio over time at various temperatures; (**d**) evolution of corrosion mass over time at various temperatures.

Figure 5a,b present the effects of ambient temperature (10 °C, 28 °C, and 45 °C) on the temporal evolution of pit diameter and pit depth under a constant salinity of 35‰. Chloride ion cyclic corrosion was employed to simulate the representative high-salinity, high-humidity, and high-temperature service conditions of the South China Sea. The results indicate that increasing temperature leads to a pronounced increase in both pit diameter and pit depth, with continuous growth observed throughout the corrosion process, demonstrating a clear accelerating effect of temperature on pitting development.

Figure 5c illustrates the time-dependent evolution of the pit diameter-to-depth ratio at different temperature levels under constant salinity. Although temperature exerts a significant influence on the pitting growth rate, the diameter-to-depth ratio under all temperature conditions gradually stabilizes during the intermediate and later stages of corrosion and eventually converges to similar values. This observation further suggests that, under the present experimental conditions, temperature primarily governs the kinetics of pitting evolution, while its influence on the geometric proportion of pits remains relatively limited.

Figure 5d compares the evolution of corrosion-induced mass loss over time at different temperatures. At the same salinity level, corrosion mass loss increases monotonically with time, and higher temperatures result in substantially greater material loss. Notably, under high-temperature conditions, some specimens experienced localized perforation and successive failure after approximately 120 min of corrosion exposure, highlighting the markedly detrimental effect of elevated temperature on structural integrity.

A comprehensive analysis indicates that increasing temperature enhances the thermal motion of ions and molecules in the electrolyte, thereby increasing the frequency of effective collisions and the probability of reaction activation. This, in turn, accelerates the kinetics of electrode processes, including electron transfer and mass transport. Such thermally activated effects substantially promote corrosion reactions, ultimately leading to the formation of deeper and larger localized pitting damage.

### 3.3. Effect of Different Temperatures and Salinity on Corrosion Pits

**Figure 6:**
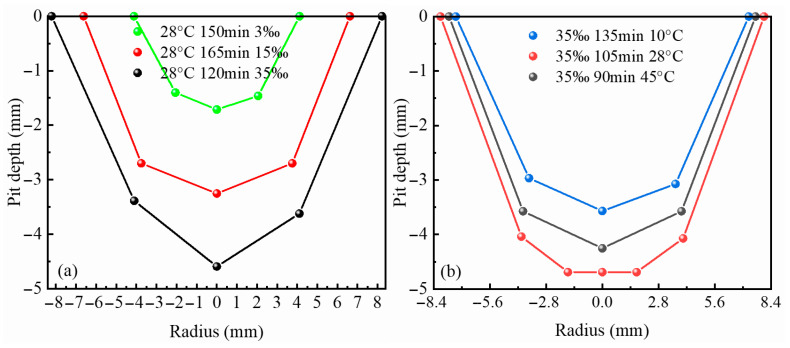
Corrosion morphology: (**a**) final morphology of measured pits at various salinities; (**b**) final morphology of measured pits at various temperatures.

Based on pit feature point data obtained using a digital probing technique, the geometric morphology and spatial evolution characteristics of corrosion pits were quantitatively characterized. Figure 6a presents representative cross-sectional morphologies of typical pits under different environmental conditions. The results indicate that, for a given corrosion duration, higher salinity leads to a pronounced increase in pit cross-sectional dimensions, demonstrating that elevated salinity significantly promotes lateral pit expansion and the overall severity of localized corrosion.

Figure 6b compares the temporal evolution of pit volume under different temperature conditions. It can be observed that increasing ambient temperature results in a substantial increase in pit volume, which continues to expand throughout the corrosion process, indicating that temperature plays a critical accelerating role in the three-dimensional development of pitting corrosion. Further analysis reveals that under coupled high-temperature and high-salinity conditions, pit growth rates are markedly higher than those governed by a single environmental factor.

Notably, several curves in the figure exhibit a convergence of corrosion depth in the peak region and its vicinity, indicating that localized pits have propagated through the specimen thickness and resulted in perforation failure. This observation suggests that under the combined effects of high temperature and high salinity, the critical transition time at which pitting corrosion evolves from a stable growth regime to a failure-dominated stage is markedly advanced, posing a severe threat to structural integrity.

## 4. Cellular Automata Finite Element Modeling

In this study, a three-dimensional Cellular Automata-Finite Element (CAFE) coupled modeling approach is proposed to simulate the evolution of stress-assisted pitting damage at the mesoscale. The model decomposes the cumulative damage evolution of the material into two strongly coupled components: localized electrochemical corrosion processes and the associated mechanical response of the material. By integrating corrosion morphology evolution with stress and temperature field distributions within a unified computational framework, the proposed model is capable of capturing the intrinsic mechanisms governing pitting development under multi-field coupling conditions.

**Figure 7:**
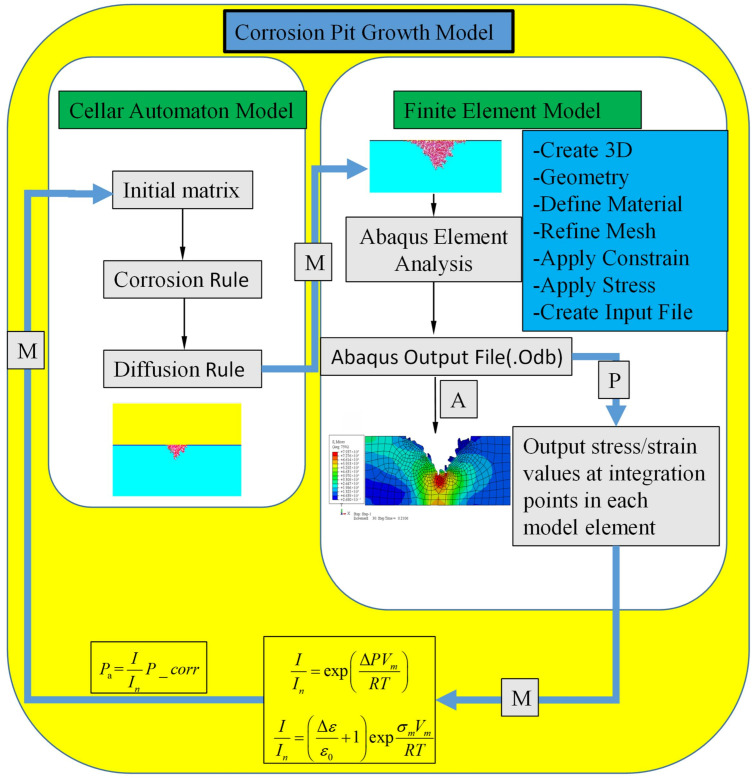
This figure presents a schematic overview of the CAFE model architecture and its developmental workflow.

During the corrosion evolution stage, a Cellular Automata (CA) approach is employed to simulate localized metal dissolution and material loss. Pitting corrosion is inherently a highly complex localized electrochemical process involving strong coupling among multiple physical, chemical, and electrochemical factors, rendering a comprehensive theoretical and computational description of all governing parameters and their interactions highly challenging. Accordingly, the proposed CAFE framework focuses on the key mechanisms that dominate pitting evolution, including the dissolution and breakdown of oxide films, electrochemical dissolution of the metallic substrate, hydrolysis of metal ions, diffusion and migration of corrosive species within the electrolyte, and the further destabilization of local passive films under multi-field interactions. Figure 7 schematically illustrates the overall architecture and modeling workflow of the CAFE framework, clearly depicting the coupling relationships among different physical processes and the pathways of information transfer. This modeling strategy enables effective mesoscale representation of the complex interactions among electrochemical reactions, mass transport, and geometric morphology evolution.

During the mechanical response phase, finite element analysis (FEA) is employed to quantitatively evaluate the local stress and temperature fields surrounding corrosion-induced pits. The coupling between the cellular automata (CA) model and the finite element model is realized through a bidirectional feedback mechanism: firstly, the evolution of cell dissolution states in the CA model is used to dynamically update the corresponding geometric features in the FEA model, thereby capturing the real-time propagation of corrosion pits; secondly, the local stress and temperature distributions obtained from FEA are fed back into the CA model to adjust the kinetic parameters governing the corrosion dissolution process, thus reflecting the influence of coupled mechanical and thermal fields on the corrosion rate.

Both analyses are executed synchronously within each time step and form a closed-loop coupling through continuous information exchange, enabling a co-evolution simulation of corrosion behavior and the material’s mechanical response. This CAFE modeling approach provides a realistic representation of pitting initiation and development under the combined effects of external loading and environmental factors, offering a robust theoretical and numerical framework for elucidating early-stage stress-assisted pitting mechanisms and the onset of environmentally assisted fracture.

### 4.1. Cellular Automata Configuration

Pitting corrosion represents one of the most prevalent and deleterious forms of degradation in steel materials. When structures are exposed over extended periods to high-salinity environments—such as coastal regions or areas subjected to road deicing salts and sediment-laden water—aggressive anions in the solution (e.g., Cl^−^) readily penetrate the protective passive film due to their small ionic radius and high mobility. This localized disruption significantly elevates the current density, further promoting cation activation and migration. Once the electric field intensity at the passive film-solution interface reaches a critical threshold, the passive film locally ruptures, initiating pitting corrosion [24] and progressively forming numerous pits that severely compromise the overall structural load-bearing capacity and integrity.

As corrosion progresses, the physicochemical environment within the pits undergoes marked changes, amplifying the electrochemical disparity between the pit interior and exterior. Localized acidification, cation enrichment, and restricted diffusion collectively exacerbate the corrosive conditions inside the pits, thereby establishing a characteristic autocatalytic corrosion mechanism that accelerates pit deepening and lateral growth [22]. Figure 1 schematically illustrates the fundamental structural features of steel pits, wherein intense electrochemical reactions occur at the local electrolyte-metal interface, continuously driving metal dissolution.

**Figure 8:**
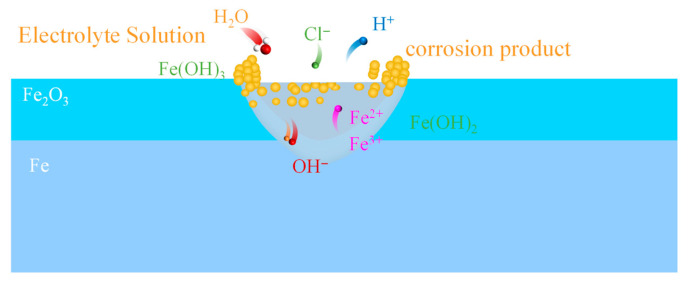
Schematic of the electrochemical reaction mechanisms involved in localized pitting corrosion.

Building on these mechanistic insights, a cellular automata (CA) model discretizes the spatial domain to abstract the local initiation, propagation, and interaction of pits, enabling mesoscale simulation of realistic pitting evolution. The model effectively captures the electrochemical behavior at the electrolyte-metal interface under high-current conditions while explicitly accounting for the formation and breakdown of the passive film. The overall system architecture is depicted in Figure 8.

**Figure 9:**
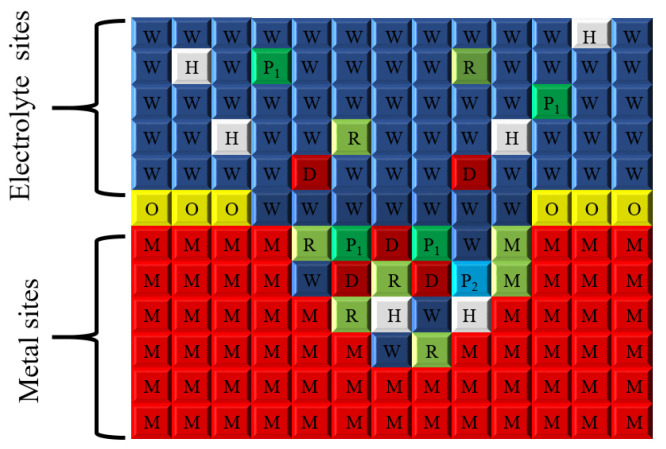
Schematic of the metric space for CA modeling.

In this study, numerical simulations were conducted on a two-dimensional lattice comprising 400 × 600 (i × j) cells, with the spatial discretization illustrated in Figure 9. The upper region of the lattice (i = 1–160) represents the aqueous environment containing corrosive ions, whereas the lower region (i = 160–360) corresponds to the metallic substrate. Considering that metal surfaces exposed to air in service environments readily form an oxide film, a passive film region of three-cell thickness (i = 160–162) was introduced at the metal-solution interface. An initial pit morphology was pre-assigned at the center of this interface to simulate the nucleation of pitting corrosion.

**Figure 10:**
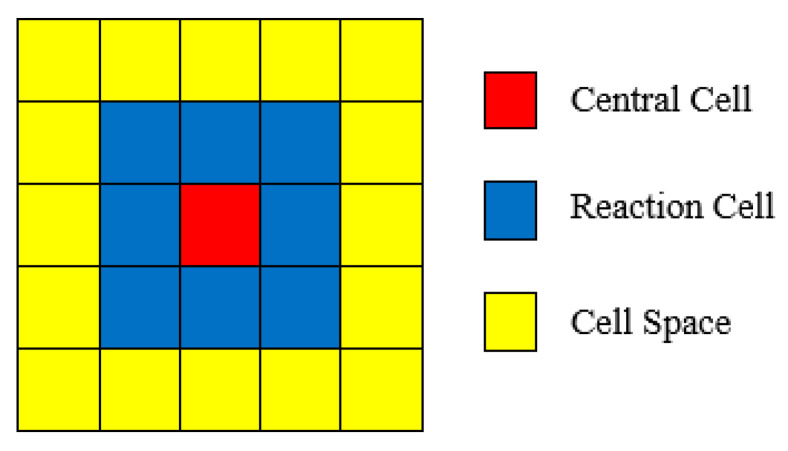
Components of a CA.

For the selection of the neighborhood structure, the model employs the Moore neighborhood, wherein the eight cells surrounding the target cell are considered (Figure 10). This neighborhood configuration provides a comprehensive representation of the multidirectional interactions inherent in electrochemical corrosion processes, making it particularly suitable for describing the diffusion of species, charge migration, and the propagation of corrosion products.

Regarding the boundary conditions, cells located at the outer edges of the metallic region were designated as non-participatory in electrochemical reactions to prevent undue influence of the computational domain boundaries on the corrosion evolution. To ensure consistency between the numerical model and experimental observations, a spatial scaling of the CA lattice was applied based on the physical depth of actual corrosion pits (approximately 5000 μm, see Figure 3). Specifically, the CA model was magnified 25-fold, such that each cell corresponds to a physical size of roughly 25 × 25 μm. Consequently, approximately 200 CA cells in the vertical direction represent the experimentally observed pit depth. The cell size was maintained constant throughout the CA simulation to ensure computational stability and comparability.

### 4.2. Electrochemical System

The cellular automata (CA) model was developed to represent an electrochemically coupled system comprising a passive film, iron, and an aqueous environment. The system simultaneously accounts for the metallic substrate, dissolved ionic species, and corrosion products in their non-hydrated forms, with iron oxide (Fe_2_O_3_) serving as a representative example. In selecting the state variables of the cellular automaton, only species exhibiting thermodynamic stability under the iron-water system at 25 °C, as indicated by the corresponding Pourbaix diagram, were included. This selection ensures electrochemical validity and physical consistency of the model.

Based on these criteria, six electrochemical species were defined as possible cell states in the model: Fe_2_O_3_, Fe, Fe^2+^, Fe^3+^, Fe(OH)_2_, and Fe(OH)_3_. The interconversion among these species is governed by the electrochemical reactions expressed in Equations (1)–(6), encompassing the principal reaction pathways involved in the corrosion of iron-based materials, including dissolution, oxidation, hydrolysis, and precipitation.

(1) $${\mathrm{Fe}}_{2}O_{3}+6H^{+}↔2{\mathrm{Fe}}^{3+}+3H_{2}O$$

(2) $${\mathrm{Fe}}^{3+}+{3H}_{2}O↔\mathrm{Fe}{\left(\mathrm{OH}\right)}_{3}↓+{3H}^{+}$$

(3) $$\mathrm{Fe}↔{\mathrm{Fe}}^{2+}+2e^{-}$$

(4) $${\mathrm{Fe}}^{2+}↔{\mathrm{Fe}}^{3+}+e^{-}$$

(5) $${\mathrm{Fe}}^{2+}+2H_{2}O↔\mathrm{Fe}{\left(\mathrm{OH}\right)}_{2}↓+2H^{+}$$

(6) $${\mathrm{Fe}}^{3+}+3H_{2}O↔\mathrm{Fe}{\left(\mathrm{OH}\right)}_{3}↓+3H^{+}$$

On this basis, Table 2 systematically enumerates the eight possible combinations of spatial positions and states that a single cell may assume within the model, thereby characterizing distinct material domains and their associated electrochemical attributes. Meanwhile, Figure 9 and Figure 10 provide schematic illustrations of these cell states and their spatial distributions, establishing a clear and unified modeling framework for subsequent analyses of corrosion morphology evolution and multiphysics coupling.

### 4.3. Evolution Rules of Cellular Automata

The CA model algorithm was implemented in the Matlab^®^ (R.2015b (8.6.0.267246)) programming environment [22]. The overall computational framework consists of two mutually coupled evolution loops. The first is the corrosion loop, which describes metal dissolution, oxidation, and the formation of corrosion products driven by electrochemical reactions. The second is the diffusion loop, which simulates the spatial migration and diffusion of various species in the solution phase during electrochemical degradation. The interaction between these two loops provides a comprehensive kinetic description of corrosion morphology evolution.

From an algorithmic perspective, the model comprises three main components: (1) an initialization module for the visualization window and computational domain, which defines the cellular lattice, initial states, and boundary conditions; (2) a core evolution module that performs stepwise updates of corrosion reactions and species diffusion; and (3) an output and post-processing module, in which simulation results are written in real time to the data file result.txt and subsequently analyzed and visualized using the data extraction script extract_data.m.

With respect to state constraints, the model strictly adheres to the single-state occupancy principle of cellular automata: at any given time and spatial location, only one cell state is permitted. For instance, sites representing distinct material phases, such as P1 and P2, cannot simultaneously occupy the same cell, thereby ensuring the physical uniqueness and logical consistency of state evolution.

#### 4.3.1. Rules for Corrosion Reactions

**Figure 11:**
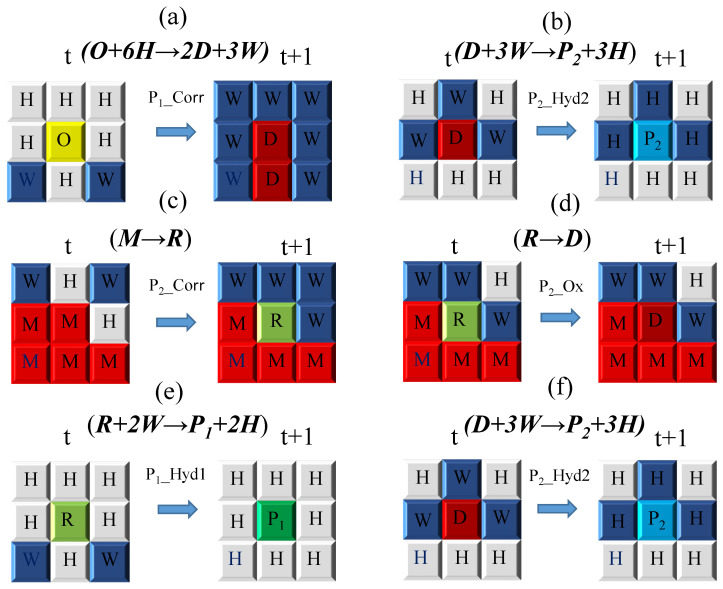
Detailed corrosion reaction rules of steel: (**a**) corrosion of iron oxide to ferric chloride; (**b**) hydrolysis of ferric chloride to ferric hydroxide; (**c**) oxidation of iron to ferrous ions; (**d**) oxidation of ferrous ions to ferric ions; (**e**) hydrolysis of ferrous ions to ferrous hydroxide; (**f**) hydrolysis of ferric ions to ferric hydroxide.

As described in Equations (1)–(6), the corrosion reaction rules are categorized into six groups. A schematic illustration of the metric space employed for CA modeling is presented in Figure 9, while Table 2 lists the corresponding cell labels associated with the various material species. The detailed corrosion reaction rules are summarized in Figure 11. Specifically, Figure 11a,b depict the corrosion reaction rules involving the oxide film layer, corresponding to Equations (1) and (2), whereas Figure 11c–f illustrate the corrosion reaction rules of the steel substrate, corresponding to Equations (3)–(6).

Here, W denotes water, H represents hydrogen ions in the aqueous phase, and O indicates iron oxide. The corresponding labels for all material species are specified in Table 2.

In Figure 11a, iron oxide (O) corrodes into ferric chloride according to Equation (1), with a corrosion probability denoted as P1Corr. This reaction is permitted when at least six neighboring sites of the iron oxide cell correspond to acidic solution sites (H). Subsequently, as illustrated in Figure 11b, the D site undergoes a hydrolysis reaction to form species (P2) in accordance with Equation (2).

As shown in Figure 11c, when at least two neighboring sites of the reactive metal (M) are acidic solution sites (H), iron (Fe) corrodes into ferrous ions, as described by Equation (3), with a corrosion probability of P2Corr. In Figure 11d, ferrous ions (R) are oxidized into ferric ions (D) following Equation (4) when at least one adjacent site is an acidic solution site (H), with an oxidation probability of P2Ox.

Figure 11e illustrates that when at least two neighboring sites of a ferrous ion (R) correspond to neutral solution sites (W), Fe^2+^ undergoes hydrolysis to form Fe(OH)_2_ according to Equation 5, with a probability of P1Hyd1. As a consequence of this reaction, the R site is replaced by P1, while the two W sites are converted into two H sites. Finally, as shown in Figure 11f, Fe^3+^ undergoes hydrolysis to form Fe(OH)_3_ in accordance with Equation (6), with a hydrolysis probability of P2Hyd2. This secondary hydrolysis reaction occurs when at least three neighboring sites of a D site are neutral solution sites (W), resulting in the replacement of the D site by P2 and the conversion of three W sites into three H sites.

#### 4.3.2. Rules for Diffusion

**Figure 12:**
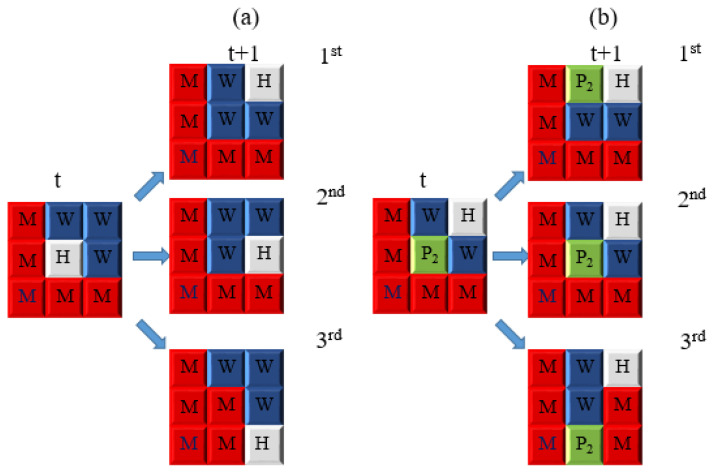
Diffusion rules associated with iron corrosion: (**a**) isotropic diffusion behavior at H sites; (**b**) precipitation tendency of iron hydroxide, with the third scenario being the most probable.

As pit growth kinetics are largely governed by diffusion processes [22], incorporating electrochemical diffusion into the simulation framework is essential. The CA model accounts for five primary diffusible species, each associated with a specific diffusion probability: Fe^2+^ (PdiffFeII), Fe^3+^ (PdiffFeIII), Fe(OH)_2_ (PdiffFeHyII), Fe(OH)_3_ (PdiffFeHyIII), and H^+^ (PdiffH). Schematic representations of the diffusion processes are shown in Figure 12a,b. Specifically, Figure 12a illustrates the diffusion of H^+^ governed by PdiffH, whereas Figure 12b depicts the diffusion of Fe(OH)_3_ controlled by PdiffFeHyIII. The diffusion mechanisms of Fe^2+^, Fe^3+^, Fe(OH)_2_, and Fe(OH)_3_ follow analogous rules within the CA framework.

In the present model, all species except Fe(OH)_2_ and Fe(OH)_3_ are assumed to undergo isotropic diffusion, meaning that they are allowed to diffuse in all directions, as illustrated in Figure 12a. By contrast, Fe(OH)_3_ is insoluble in water, whereas Fe(OH)_2_ may exist either as a dissolved species or as a readily oxidizable solid precipitate, as shown in Figure 12b. For these insoluble hydroxides, the model introduces a downward migration probability induced by gravity, thereby enhancing the tendency of corrosion products to accumulate in lower regions of the computational domain.

This gravitational effect is incorporated into the CA framework through a precipitation factor, denoted as Sed. The factor modifies the diffusion behavior of Fe(OH)_2_ and Fe(OH)_3_ by scaling their diffusion probabilities within the diffusion loop. Consequently, the model not only captures the deposition of hydroxides but also accounts for the reduction in deposition rates under flowing conditions, providing a more comprehensive description of corrosion product behavior.

The precipitation factor therefore plays a dual role: it characterizes hydroxide deposition while simultaneously reflecting the diminished deposition rate in the presence of fluid flow. In addition, the displacement of corrosive media is explicitly simulated to represent their more dynamic behavior relative to other diffusible species. This approach can be readily extended to additional diffusion entities, including [O], [H], [R], [D], and [P]. The corrosion, hydrolysis, and diffusion probabilities assigned to all species in the CA model are summarized in Table 3.

### 4.4. Finite Element Modeling

#### 4.4.1. Finite Element Study of Pit Geometry

Finite element analysis (FEA) was employed to evaluate the local stress and temperature fields in the vicinity of pitting cavities generated by the cellular automata (CA) model. Preliminary calculations indicate that, over up to ten consecutive CA time steps, the geometric evolution of the pitting cavity is minimal, with the overall morphology remaining essentially unchanged. Owing to this characteristic, it is unnecessary to recompute the stress and temperature fields at every CA time step, allowing for a substantial reduction in computational cost without compromising accuracy.

Accordingly, a strategy was adopted in which finite element analyses were performed every five CA time steps to update the stress and temperature distributions around the pit. To enable efficient interaction between the CA model and the finite element solver, an automated coupling algorithm was developed using Matlab^®^ scripts, facilitating bidirectional transfer of pit geometry, material parameters, and computational results between the CA framework and the FEA module.

**Figure 13:**
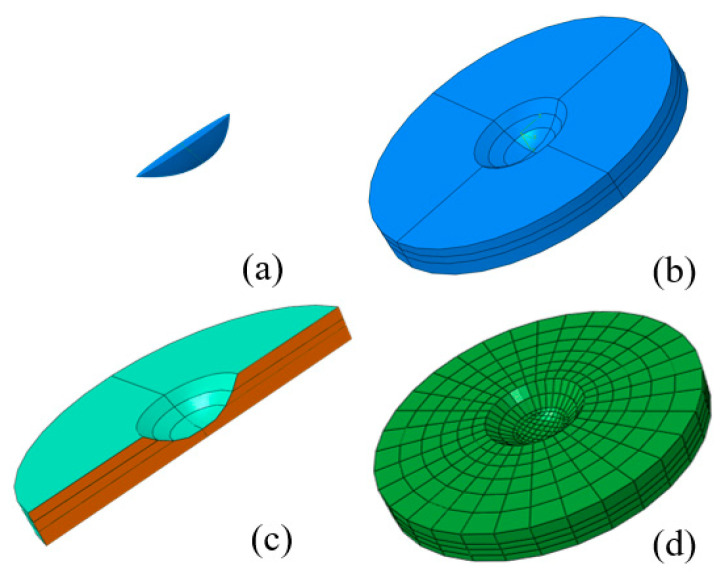
Construction of a 3D corrosion pit model: (**a**) 3D pit geometry; (**b**) Boolean operation and pit modeling; (**c**) planar solid sectioning; (**d**) isolated mesh generation.

**Figure 14:**
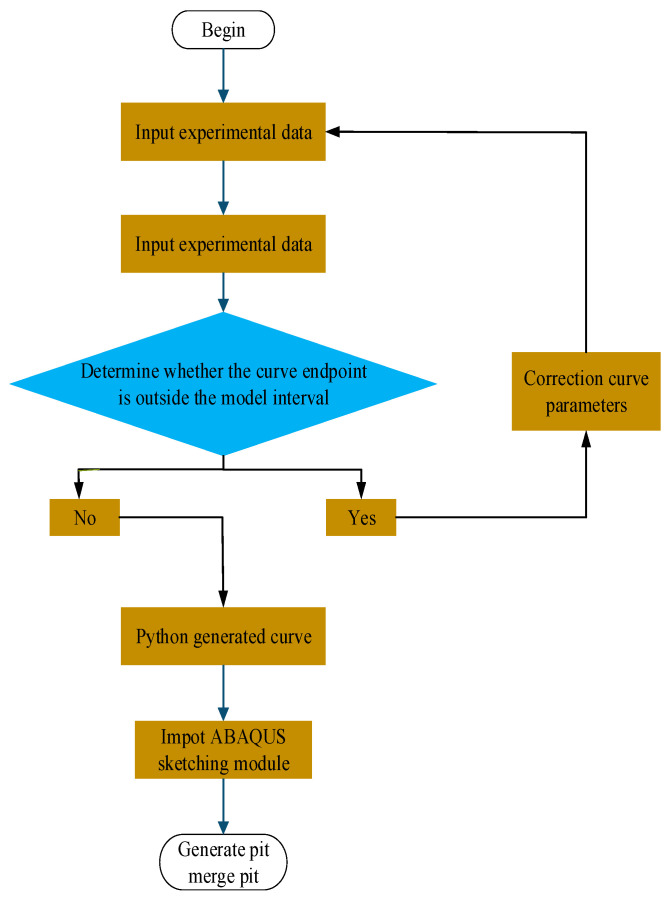
Flow chart illustrating the complete process of 3D corrosion pit model construction.

Furthermore, the pit geometries predicted by the CA model were refined based on experimental measurements, and the corrected pit profiles were parameterized using a quadratic parabolic function. On this basis, a three-dimensional modeling program was developed in Python (ABAQUS 6.14-Python 2.7) to import the fitted curves and generate the three-dimensional morphology of the pitting cavity. As illustrated in Figure 13a, the constructed pit geometry was combined with a circular steel plate model through Boolean operations, yielding the complete pit structure shown in Figure 13b. Figure 14 presents a schematic overview of the entire workflow for pit geometry construction and coupled finite element analysis.

Under experimental conditions of a salinity of 42‰ and a temperature of 35 °C, the representative pitting cavity exhibits a depth of approximately 3.7 mm and a mouth diameter of approximately 18 mm.

The longitudinal cross-section of the generated pitting cavity exhibits an overall quadratic profile, which inevitably introduces a geometric inflection point during three-dimensional reconstruction. If not treated properly, this inflection point may lead to numerical singularities in finite element modeling and solution procedures, potentially resulting in non-convergence or simulation failure. To ensure geometric smoothness and computational robustness, an additional planar section was introduced at the inflection location (see Figure 13c), thereby effectively eliminating the adverse effects of geometric discontinuities on numerical stability.

To simplify the model and improve computational efficiency, the influence of the zinc coating with a thickness of approximately 8 μm was neglected in the finite element analysis. The primary function of the zinc layer is to enhance the atmospheric corrosion resistance of steel plates; however, under high-current-density conditions, the coating itself also undergoes electrochemical dissolution. Given that the coating thickness is negligible compared to the approximately 5000 μm thickness of the steel substrate, and that the surface film was mechanically removed prior to experimentation, its impact on pit morphology evolution and the overall mechanical response is minimal. Furthermore, comparative electrochemical corrosion experiments conducted on galvanized and non-galvanized steels revealed no significant differences in macroscopic corrosion behavior, observable phenomena, or experimental data. In addition, explicitly incorporating the coating as a composite layer in the finite element model would substantially increase the complexity of constitutive definitions, interfacial contact relations, and mesh generation. For these reasons, the zinc coating was not explicitly considered in the present modeling framework.

For the axisymmetric circular geometry, the model was partitioned twice in the radial direction to construct a symmetric sector domain, facilitating the generation of regular, high-quality meshes and enhancing numerical stability and convergence. Along the thickness direction, the mesh was discretized into at least four layers, with local refinement applied near the surface and the pit center to accurately capture stress and temperature gradients. The overall mesh was generated using a sweep technique, resulting in a structured hexahedral mesh comprising a total of 4127 elements, as shown in Figure 13d.

It should be noted that excessive mesh refinement does not necessarily improve computational accuracy but instead increases computational cost, whereas overly coarse meshes may lead to numerical non-convergence and an inadequate representation of local field variables. Balancing accuracy and efficiency, Q3D8 thermo-electro-mechanical coupled elements were employed in this study, which are well suited to capturing the localized multiphysics responses associated with pitting corrosion.

#### 4.4.2. Electro-Thermo-Mechanical Coupled Constitutive Theory

According to electrodynamics, the electric current continuity equation in metallic materials can be expressed as:

(7) $$\nabla_{\bar{x}}\cdot \left(j+\frac{\partial D}{\partial t}\right)=0$$

Here, j denotes the electric current density, D represents the electric displacement vector, and ∇ is the Hamilton (nabla) operator, defined as:

(8) $$\nabla_{\bar{x}}=\frac{\partial }{\partial x_{i}}{\hat{e}}_{i}$$

Equation (7) can be reformulated using the following relations:

(9) $$j=\sigma_{E}E$$

(10) $$D=\varepsilon_{E}E$$

(11) $$E=-\nabla_{\bar{x}}\phi$$

where *E* denotes the electric field intensity, $\sigma_{E}$ is the electrical conductivity of the material, $\varepsilon_{E}$ represents the dielectric permittivity, and ϕ is the electric potential.

After reformulation, the equation can be expressed in terms of the electric potential as:

(12) $$\nabla_{\bar{x}}\cdot \left(\sigma_{E}\nabla_{\bar{x}}\phi +\varepsilon_{E}\nabla_{\vec{x}}\frac{\partial \phi }{\partial t}\right)=0$$

Dirichlet Boundary Condition:

(13) $$\phi =\phi_{d}$$

Impedance Boundary Condition:

(14) $$\hat{n}\cdot \sigma_{E}\nabla_{\bar{x}}\phi =\frac{\phi }{RS}$$

According to Fourier’s law of heat conduction:

(15) $$\frac{\partial q}{\partial t}=-\kappa \nabla_{\vec{x}}T$$

where q denotes the heat flux density, κ is the thermal conductivity, and T represents the temperature.

Derived heat conduction equation:

(16) $$\rho c_{p}\frac{\partial T}{\partial t}=\nabla_{\bar{x}}\cdot \left(\kappa \nabla_{\vec{x}}T\right)+Q+\nabla_{\vec{x}}\cdot \left(\sigma_{ij}\cdot v_{i}\right)$$

Here, ρ denotes the material density, $c_{p}$ is the specific heat capacity, and Q represents the volumetric heat generation rate.

Dirichlet Boundary Condition:

(17) $$x_{3}=0\;\Rightarrow \;T=T_{d}$$

Air Convection Boundary Condition:

(18) $$x_{3}=d\;\Rightarrow \;\hat{n}\cdot \left(\kappa \nabla_{\bar{x}}T\right)=-h\left(T-T_{a}\right)$$

According to linear thermoelastic theory, the infinitesimal stress-displacement relationship can be expressed as:

(19) $$\varepsilon_{ij}=\frac{1}{2}\left(\frac{\partial u_{i}}{\partial x_{j}}+\frac{\partial u_{j}}{\partial x_{i}}\right)$$

where ε denotes the strain tensor, u represents the displacement vector, and i=j=1,2,3.

The stress–strain constitutive relationship is given by:

(20) $$\sigma_{ij}=C_{ijkl}\left(\varepsilon_{kl}-\alpha \delta_{kl}\Delta T\right)$$

where $\sigma_{ij}$ is the stress tensor, $C_{ijkl}$ denotes the elastic stiffness matrix, α is the coefficient of thermal expansion, $\delta_{kl}$ is the Kronecker delta symbol, and $\Delta T=T-T_{0}$ represents the temperature increment relative to the reference configuration.

Here, the following relations hold:

(21) $$C_{ijkl}=\lambda \delta_{ij}\delta_{kl}+\mu \left(\delta_{ik}\delta_{jl}+\delta_{il}\delta_{jk}\right)$$

where λ and μ are the Lame constants.

Here, $\delta_{kl}$ denotes the Kronecker delta, given by:

(22) $$\delta_{ij}=\left\{\begin{array}{cc} 1 & i=j\\ 0 & i\neq j \end{array}\right.$$

According to the conservation of momentum:

(23) $$\rho \frac{\partial v_{i}}{\partial t}=\frac{\partial \sigma_{ij}}{\partial x_{j}}+f_{i}$$

where ρ denotes the material density, v represents the velocity vector, and $f_{i}$ is the body force per unit volume.

By combining Equations (19), (20) and (23), the governing equation for thermoelastic stress can be obtained as:

(24) $$\frac{1}{2}\frac{\partial }{\partial x_{j}}\left[C_{ijkl}\left(\frac{\partial u_{k}}{\partial x_{l}}+\frac{\partial u_{l}}{\partial x_{k}}\right)\right]=\frac{\partial }{\partial x_{j}}\left(C_{ijkl}\alpha \delta_{kl}\Delta T\right)-f_{i}$$

Displacement boundary condition:

(25) $$u_{i}=u_{d}$$

Owing to the strong coupling among the electrical, thermal, and mechanical fields, deriving an analytical solution is generally intractable. Consequently, numerical methods are adopted to solve the coupled governing equations. Here, the finite element method is utilized to discretize the electro-thermal-mechanical field equations into algebraic form, allowing for efficient and robust numerical implementation.

The thermo-mechanical coupling formulation is first constructed as:

(26) $$\left\{u\right\}=\left[N\right]\left\{q\right\}$$

where the interpolation functions are defined as:

(27) $$\left[N\right]=\left[\begin{array}{cccccccc} N_{1} & 0 & 0 & \ldots & N_{i} & 0 & 0 & \ldots \\ 0 & N_{1} & 0 & \ldots & 0 & N_{i} & 0 & \ldots \\ 0 & 0 & N_{1} & \ldots & 0 & 0 & N_{i} & \ldots \end{array}\right]$$

From the infinitesimal strain-displacement relationship:

(28) $$\left\{\varepsilon \right\}=\left[D\right]\left\{u\right\}=\left[D\right]\left[N\right]\left\{q\right\}=\left[B\right]\left\{q\right\}$$

where the differential operator is defined as:

(29) $$\left[D\right]=\left[\begin{array}{cccccc} \frac{\partial }{\partial x_{1}} & 0 & 0 & \frac{\partial }{\partial x_{2}} & 0 & \frac{\partial }{\partial x_{3}}\\ 0 & \frac{\partial }{\partial x_{2}} & 0 & \frac{\partial }{\partial x_{1}} & \frac{\partial }{\partial x_{3}} & 0\\ 0 & 0 & \frac{\partial }{\partial x_{3}} & 0 & \frac{\partial }{\partial x_{2}} & \frac{\partial }{\partial x_{1}} \end{array}\right]$$

where the stress–strain relationship can be expressed in matrix form as:

(30) $$\varepsilon ={\left\{\begin{array}{cccccc} \varepsilon_{x_{1}} & \varepsilon_{x_{2}} & \varepsilon_{x_{3}} & \gamma_{x_{1}x_{2}} & \gamma_{x_{2}x_{3}} & \gamma_{x_{3}x_{1}} \end{array}\right\}}^{T}$$

(31) $$\sigma ={\left\{\begin{array}{cccccc} \sigma_{x_{1}} & \sigma_{x_{2}} & \sigma_{x_{3}} & \tau_{x_{1}x_{2}} & \tau_{x_{2}x_{3}} & \tau_{x_{3}x_{1}} \end{array}\right\}}^{T}$$

From the stress–strain constitutive relationship:

(32) $$\left\{\sigma \right\}=\left[E_{c}\right]\left(\left\{\varepsilon \right\}-\left\{\varepsilon_{T}\right\}\right)=\left[E_{c}\right]\left[B\right]\left\{q\right\}-\left[E_{c}\right]\left\{\varepsilon_{T}\right\}$$

where the strain induced by temperature change is given by:

(33) $$\varepsilon_{T}={\left\{\begin{array}{cccccc} \alpha \Delta T & \alpha \Delta T & \alpha \Delta T & 0 & 0 & 0 \end{array}\right\}}^{T}$$

Constitutive matrix:

(34) $$\left[E_{c}\right]=\left[\begin{array}{cccccc} \lambda +2\mu & \lambda & \lambda & 0 & 0 & 0\\ \lambda & \lambda +2\mu & \lambda & 0 & 0 & 0\\ \lambda & \lambda & \lambda +2\mu & 0 & 0 & 0\\ 0 & 0 & 0 & \mu & 0 & 0\\ 0 & 0 & 0 & 0 & \mu & 0\\ 0 & 0 & 0 & 0 & 0 & \mu \end{array}\right]$$

where the Lame constants are defined as:

(35) $$\lambda =\frac{vE}{\left(1+v\right)\left(1-2v\right)}$$

(36) $$\mu =\frac{E}{2\left(1+v\right)}$$

where E denotes Young’s modulus and v is Poisson’s ratio.

By applying the finite element method to Equations (26), (28) and (32), a matrix equation can be obtained as:

(37) $$\left[K\right]\left\{q\right\}=\left\{f_{e}\right\}+\left\{f_{T}\right\}$$

where

(38) $$\left[K\right]=\int_{V}{\left[B\right]}^{T}\left[E_{c}\right]\left[B\right]dV$$

(39) $$\left\{f_{e}\right\}=\int_{V}[N{]}^{T}\left\{f_{V}\right\}dV+\int_{S}[N{]}^{T}\left\{f_{S}\right\}dS$$

(40) $$\left\{f_{T}\right\}=\int_{V}{\left[B\right]}^{T}\left[E_{c}\right]\left\{\varepsilon_{T}\right\}dV$$

where $\left\{f_{S}\right\}$ denotes the surface traction and $\left\{f_{V}\right\}$ represents the body force.

The Von Mises yield criterion is adopted to evaluate the bonding strength of the material:

(41) $$\sigma_{\mathrm{Von}}=\left\{\frac{1}{2}\left[{\left(\sigma_{x_{1}}-\sigma_{x_{2}}\right)}^{2}+{\left(\sigma_{x_{2}}-\sigma_{x_{3}}\right)}^{2}+{\left(\sigma_{x_{3}}-\sigma_{x_{1}}\right)}^{2}\right]+3{\left(\tau_{x_{1}x_{2}}^{2}+\tau_{x_{2}x_{3}}^{2}+\tau_{x_{3}x_{1}}^{2}\right)}^{\frac{1}{2}}\right.$$

#### 4.4.3. Rules for Mechanical-Electrochemical Effect

Previous studies have demonstrated that the anodic dissolution behavior of metals is significantly activated under mechanical deformation [22,24]. Meanwhile, the presence of an externally applied current further accelerates the growth rate of corrosion pits [23,24]. Within the theoretical framework of the Gutmann model [22,24], a kinetic model is therefore established in this study to describe anodic dissolution of metals subjected to the combined effects of elastic and plastic deformation.

The model predictions indicate that elastic deformation exerts a relatively limited influence on the corrosion rate. In contrast, once plastic deformation is initiated, the local anodic dissolution rate increases markedly, with this enhancement becoming particularly pronounced during strain hardening. As indicated by Equation (44), an increase in current density directly amplifies the electrochemical driving force, thereby intensifying the corrosion process. Simultaneously, thermally induced plastic deformation arising from the coupled effects of stress and strain further magnifies this acceleration effect.

(42) $$\frac{I}{I_{n}}=\exp\left(\frac{\Delta PV_{m}}{RT}\right)$$

(43) $$\frac{I}{I_{n}}=\left(\frac{\Delta \varepsilon }{\varepsilon_{0}}+1\right)\exp\frac{\sigma_{m}V_{m}}{RT}$$

(44) $$P_{a}=\frac{I}{I_{n}}P\_corr$$

where *I* denotes the anodic current induced by mechanical deformation, *I_n_* is the anodic current in the undeformed state, Δ*P* represents the hydrostatic pressure increment, *V_m_* is the molar volume, Δ*ε* is the equivalent plastic strain, *ε*_0_ denotes the strain at the onset of strain hardening, *σ_m_* is the hydrostatic stress, *R* is the universal gas constant, and *T* is the absolute temperature. Equations (42)–(44) together constitute a characteristic positive feedback regulation mechanism: material loss induced by electrochemical corrosion and mechanical deformation alters the local stress–strain state, while the enhanced plastic deformation in turn promotes anodic dissolution. As a result, the corrosion evolution is continuously accelerated under the coupled action of electrical, thermal, and mechanical fields.

According to the Gutmann model, the hydrostatic pressure and equivalent plastic strain at each point on the pit surface, obtained from finite element analysis, are used to evaluate the influence factor I/In. This factor exceeds unity in regions of the metal substrate subjected to elevated local stress and plastic deformation. At a temperature of 25 °C, the normalized constants adopted in the model are T = 298 K, V_m_ = 7.0923 × 10^−6^ m^3^mol^−1^ (for iron), and R = 8.314 m^2^kgs^−2^K^−1^mol^−1^. The strain corresponding to the onset of strain hardening (i.e., yielding) is taken as 0.2%.

The influence factors for all elements in the finite element model are systematically evaluated. Since each finite element can be mapped onto a corresponding cell in the CA model, the effect of stress on corrosion is incorporated by modifying the lattice state of metal cells within the CA framework. Specifically, a stress-dependent influence factor is introduced to adjust the corrosion probability P_Corr_,_ thereby enabling a coupled representation of mechanical behavior and corrosion evolution.

#### 4.4.4. Constitutive Parameters

In the finite element analysis (FEM), the electro-thermo-mechanical-chemical (ETMC) multiphysics coupling model is strictly configured and calibrated in accordance with the experimental conditions. Given that the experimental material is galvanized pure iron, iron material properties are adopted in the finite element model. Owing to the extremely small thickness of the zinc coating and its removal prior to the experiments by mechanical polishing, its influence on the overall electro-thermal-mechanical coupling behavior is considered negligible and is therefore not explicitly included in the numerical model.

All simulations are conducted using the kg-mm-s unit system. The material and physical parameters are specified as follows. The thermal conductivity of iron is treated as temperature-dependent, decreasing from 0.0762 to 0.07497 over the temperature range of 20–100 °C. The density is set to 7.87 × 10^−6^kg/mm^3^. The elastic modulus and Poisson’s ratio are taken as 210,000 MPa and 0.291, respectively. The plastic constitutive behavior is determined based on uniaxial tensile test data of Q235 steel, where a stress of 235 MPa corresponds to zero plastic strain and 350 MPa corresponds to a plastic strain of 0.05. The electrical conductivity is also defined as temperature-dependent, decreasing from 11,236 to 8666 over the temperature range of 20–100 °C. The coefficient of thermal expansion increases from 1.22 × 10^−5^ to 1.5 × 10^−5^ within the temperature range of 20–150 °C. The Joule heat conversion factor is set to unity, assuming no heat exchange with the external environment. The specific heat capacity of iron is taken as 440 J/(kg·K).

Additional simulation settings are specified as follows. A coupled thermo-electrical structural analysis step is employed, and nonlinear transient calculations are performed with a total simulation duration of 900 s (15 min). A fixed time-stepping strategy is adopted, with an initial increment of 0.01 s, a minimum increment of 0.00001 s, and a maximum increment of 100 s. Meanwhile, the maximum allowable temperature change per increment is restricted to 0.3 °C. A hydrostatic pressure of 0.035 MPa is applied to the circular metal surface. An equivalent concentrated current of 2.06 A is imposed on the pit surface, with the current magnitude smoothly ramped up over time. Displacement constraints are applied along the periphery of the circular steel plate, and a reference point is defined at the center of the bottom surface, where the electric potential is fixed at 0 V. The initial ambient temperature field is uniformly set to 30 °C.

The above multiphysics parameters and boundary conditions are fully consistent with the experimental setup, thereby providing a reliable basis for subsequent finite element investigations of pitting corrosion evolution under coupled electro-thermo-mechanical-chemical interactions.

### 4.5. Optimization of CAFE Model

A major advantage of the CAFE model lies in its capability to realistically and continuously capture the time-dependent evolution of pitting corrosion geometry while simultaneously providing a quantitative description of the local stress distribution in the vicinity of corrosion pits. However, the predictive accuracy of the model strongly depends on the appropriate selection and calibration of its input parameters.

To achieve effective parameter calibration, numerical simulation results must be systematically compared with experimentally measured pitting growth data. Given that the model involves multiple mutually coupled input parameters, the associated error function typically exhibits strong nonlinearity and a highly multimodal landscape in the parameter space. Under such circumstances, empirical parameter selection alone is insufficient to guarantee a globally optimal solution. It is therefore necessary to introduce an optimization strategy to determine, in a probabilistic sense, an optimal set of input parameters that minimizes the discrepancy between numerical predictions and experimental observations over the entire parameter space.

From the perspective of pitting corrosion modeling, the maximum pit depth is one of the most critical geometric descriptors of pit evolution and damage severity, and it is also among the most widely adopted evaluation metrics in engineering practice. Accordingly, the time-dependent mean squared error (MSE) between the simulated and experimentally measured maximum pit depths is selected as the objective function for optimization. By minimizing this objective function, the parameters of the CA model are effectively calibrated and refined.

Building upon this calibrated framework, the optimized CA model is further coupled with finite element analysis to investigate stress-assisted pitting corrosion growth. This integrated approach provides a robust computational framework for elucidating the influence of external mechanical loading on pitting corrosion evolution mechanisms.

## 5. Modeling Results and Discussion

This section provides an in-depth discussion of the numerical simulation results obtained using the CAFE model, which has been calibrated against experimental data on the actual time scale. First, the corrosion morphology evolution predicted by the cellular automaton module is presented, including the temporal variations in pit diameter, depth, and overall geometric shape. Subsequently, the influence mechanisms of key parameters-such as corrosion rate, precipitation factor, and metal-ion hydrolysis probability-on pitting corrosion evolution are systematically analyzed from a mechanistic perspective. Finally, the results of electro-thermo-mechanical-chemical multiphysics finite element simulations are reported and compared with corresponding experimental observations, thereby assessing the accuracy and reliability of the model in predicting pitting corrosion behavior under complex service conditions.

### 5.1. Evolution of Pit Characteristics with Time

**Figure 15:**
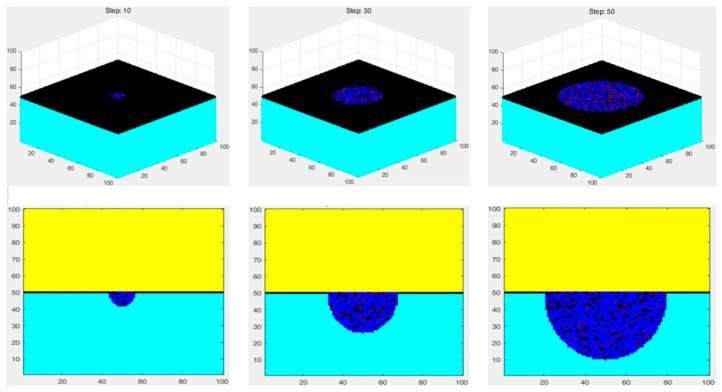
Three-dimensional morphologies of pitting corrosion at different time steps.

The fundamental evolution principle of the three-dimensional cellular automaton (3D CA) model remains consistent with that of the two-dimensional case, while an additional spatial dimension is incorporated at the algorithmic implementation level. Prior to initiating the 3D CA algorithm, a triple-loop framework is first constructed to initialize the corrosion system, including the electrolyte solution layer (highlighted in yellow in Figure 15), the passive film layer (indicated by thick black lines), and the initial geometric morphology of the pitting corrosion cavity.

With respect to spatial parameterization, the center position of horizontal corrosion propagation is defined as j = 300, with an initial horizontal expansion range of 50 cells and a corresponding evolution duration of 50 time steps. In the vertical direction, the corrosion propagation range is confined between imin = 160 and imax = 360, and the associated evolution time is likewise set to 50 time steps. Meanwhile, a mapping relationship between the CA time steps and the actual experimental time scale is established. The temporal evolution of pit diameter and depth is formulated as time-dependent binding functions, which may be expressed either in a linear form or, alternatively, represented by power-law functions fitted to experimental data. This formulation enhances the model’s capability to capture the inherently nonlinear characteristics of pitting corrosion evolution.

To provide a clear visualization of the three-dimensional evolution of pitting corrosion morphology at different time steps, Figure 15 presents representative 3D simulation results at selected stages. As can be clearly observed, the pit diameter, depth, and overall geometric configuration evolve continuously with time. As the corrosion process progresses, the passive film undergoes a cyclic sequence of dissolution, repassivation, and subsequent dissolution.

To accurately capture this dynamic behavior, interface-tracking and state-transition functions are incorporated into the model to describe the formation and breakdown of the passive film under electrochemical interactions. In Figure 15, discontinuously distributed black regions can be observed at the interface between the cyan-colored metal substrate and the corrosion products, corresponding to the evolving passive film layer.

### 5.2. Impact of Corrosion and Diffusion on Pit Propagation

Localized corrosion is intrinsically a highly complex stochastic evolutionary process, whose kinetics are governed by the interplay of multiple coupled physical and chemical mechanisms. These mechanisms include anodic dissolution of the metal substrate, diffusion and hydrolysis of corrosion species in the electrolyte, as well as the dissolution and repassivation of the passive film. Within this framework, a cellular automata (CA) model-whose parameters were optimized under simulated flow conditions-was employed to systematically investigate the key controlling factors governing the evolution of localized corrosion.

Specifically, the temporal evolution of the number of dissolved cells was analyzed with respect to three dominant mechanisms: (i) metal oxidation leading to the formation of Fe^2+^, (ii) hydrolysis of Fe^3+^ species, and (iii) the deposition and accumulation of corrosion products, namely Fe(OH)_2_ and Fe(OH)_3_, within the corrosion pit. By independently varying the parameters associated with these three processes while keeping all other model parameters listed in Table 3 unchanged, the relative contributions of each physicochemical mechanism at different stages of corrosion evolution were quantitatively assessed, thereby elucidating their respective roles in governing localized corrosion kinetics.

#### 5.2.1. Influence of Oxidation of Metal

Activation-controlled corrosion processes have been widely recognized to be primarily governed by the rate at which metal atoms are oxidized into cations capable of participating in redox reactions. Extensive experimental evidence demonstrates that under service conditions characterized by elevated temperature, high salinity, and high humidity, the mobility of cations in the electrolyte is significantly enhanced, thereby accelerating the overall kinetics of electrochemical corrosion. Based on these considerations, a corrosion probability, P_Corr, is introduced in the present model to characterize the oxidation rate of iron (Fe) to Fe^2+^, enabling a quantitative description of metal dissolution under activation-controlled conditions.

**Figure 16:**
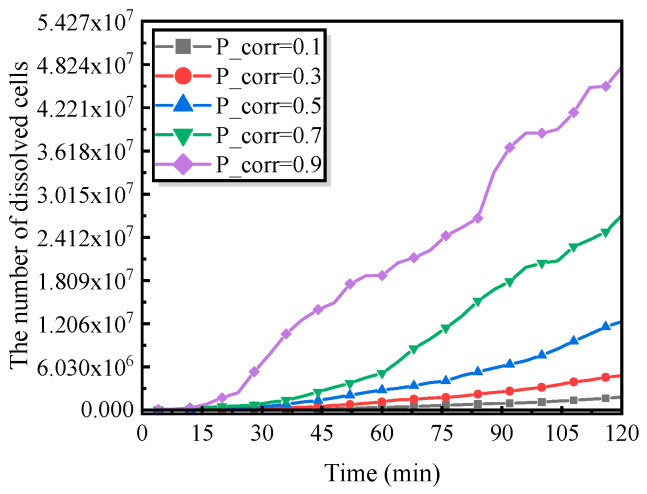
Effect of corrosion probability.

As shown in Figure 4, the corrosion mass exhibits a characteristic power-law dependence on time, an empirical relationship that has been extensively validated in experimental studies of pitting corrosion [22] and is widely adopted to describe the pit growth kinetics of steels [22]. Further evidence from Figure 16 indicates that, within the same time interval, the number of dissolved cells increases markedly with increasing P_Corr. A higher corrosion probability signifies a stronger tendency for metal oxidation, leading to elevated local concentrations of active corrosion species such as Fe^2+^ within the pit. This, in turn, accelerates metal dissolution and promotes the growth and lateral expansion of pitting corrosion.

From a physical perspective, variations in P_Corr collectively reflect the intensifying effects of high temperature, high salinity, high humidity, and high current density on corrosion kinetics. These results not only reveal the pronounced time-dependent nature of localized corrosion damage but also underscore the dominant role of metal dissolution kinetics in governing the evolution of pitting corrosion.

#### 5.2.2. Influence of Accumulation of Corrosion Products

As indicated by the experimental observations (Figure 2), the transport of corrosion products from the interior of a pit to the surrounding environment plays a critical role in determining whether pitting corrosion can sustain stable growth. To capture this effect, an accumulation factor, Sed, is introduced in the model to quantify the deposition and accumulation rate of corrosion products, specifically Fe(OH)_2_ and Fe(OH)_3_, within the pit during localized dissolution.

**Figure 17:**
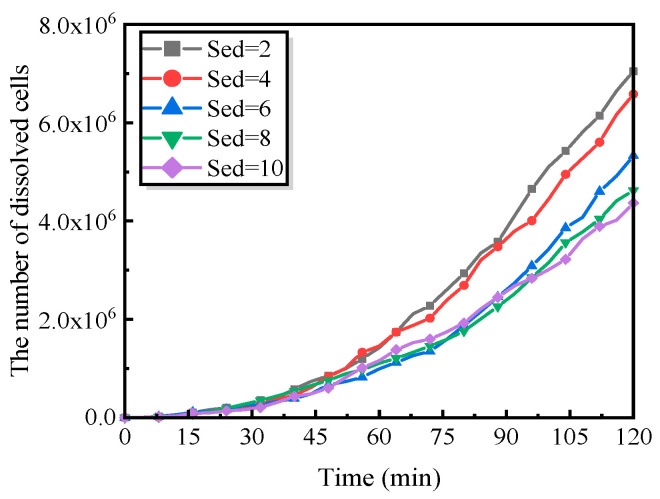
Effect of precipitation coefficient.

Figure 17 presents the temporal evolution of the number of dissolved cells under different values of Sed. The results show that the overall corrosion extent increases monotonically with time, in good agreement with the experimental trends. However, at a given time scale, higher Sed values correspond to a markedly reduced number of dissolved cells, indicating that rapid accumulation of corrosion products effectively suppresses metal dissolution. In contrast, lower Sed values represent enhanced mass transport of corrosion products away from the pit, leading to increased metal loss, accelerated pit growth, and a pronounced intensification of the overall damage evolution.

From a mechanistic perspective, the persistent accumulation of corrosion products within the pit can impose diffusion-controlled limitations on the corrosion process by forming an effective transport barrier between reactive species [22]. In practical engineering systems, a reduced Sed value may arise primarily from two scenarios: (i) increased fluid flow velocity, which efficiently removes corrosion products from the pit interior, thereby reducing deposition, enhancing mass transport, and promoting electrochemical reactions and metal dissolution; and (ii) localized acidification within the pit induced by metal-ion hydrolysis, which increases the solubility of corrosion products and consequently diminishes their accumulation and precipitation rates.

#### 5.2.3. Influence of Metal Ion Hydrolysis

**Figure 18:**
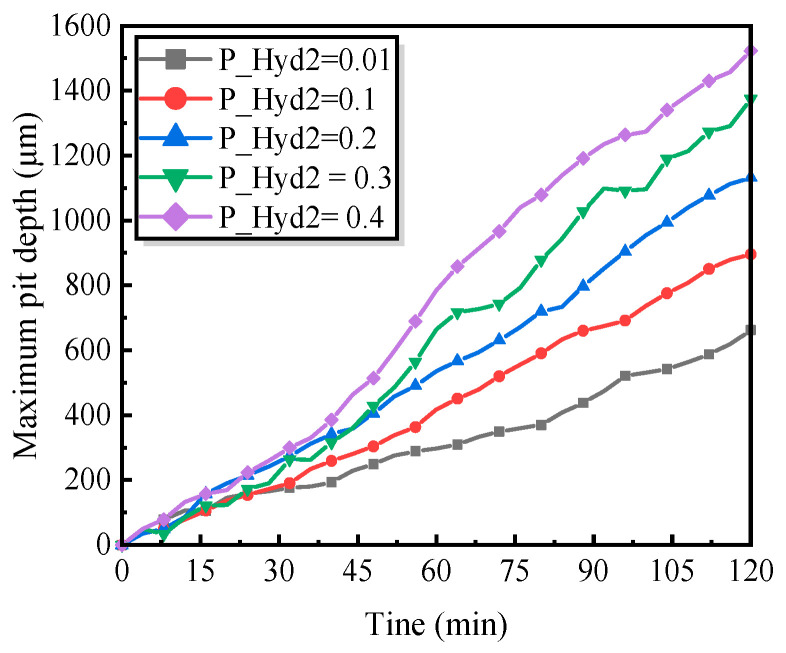
Corrosion depth vs hydrolysis probability.

To systematically assess the influence of hydrolysis probability on the evolution of pit depth, a series of comparative numerical simulations was performed. As shown in Figure 18, when the hydrolysis probability P_Hyd2 reaches its maximum value, the overall corrosion damage—characterized by both the maximum pit depth and the total metal loss—also attains its highest level.

This behavior can be attributed to two synergistic mechanisms. First, an increased hydrolysis probability promotes the continuous generation of acidic active sites, accelerating the acidification of the pit interior and thereby significantly enhancing the anodic dissolution rate of the metal, which in turn accelerates pit deepening. Second, the progressive accumulation of hydrogen ions intensifies the dissolution of the passive film, further amplifying the metal dissolution rate and sustaining the rapid growth of the pitting corrosion.

During pit evolution, the competition between the two mechanisms described above jointly governs the pit growth rate and its evolutionary trajectory. Notably, as P_Hyd2 increases from 0.01 to 0.4, the enhanced hydrolysis kinetics markedly accelerate the formation of corrosion products. With the progressive accumulation of these products within the pit, the local pH may temporarily increase at certain stages. However, the continued hydrolysis of the salt film subsequently releases additional hydrogen ions, driving the solution environment back toward acidification.

As indicated by Equation (6), an elevated hydrogen ion concentration directly accelerates the dissolution of the passive film, thereby reinforcing localized metal dissolution and ultimately intensifying the overall pitting corrosion process.

### 5.3. Meshing

To avoid nonphysical numerical artifacts in the electro-thermo-mechanical-chemical coupled analysis, appropriate control of the finite element mesh resolution is essential. Specifically, the characteristic size of solid elements should not be smaller than the representative scale of key geometric features-such as corrosion pits, particles, or local structural units-to ensure both numerical accuracy and physical consistency of the simulation results.

**Figure 19:**
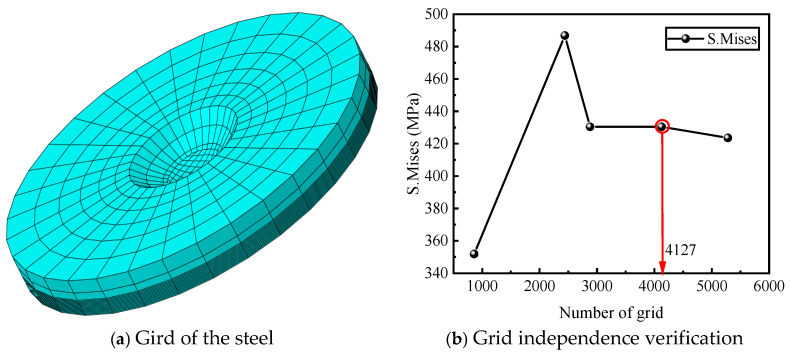
Finite element mesh discretization and mesh-independence validation.

Figure 19a illustrates the finite element mesh configurations of a perforated steel plate under different computational domain sizes, demonstrating that variations in domain scale markedly influence mesh characteristics and element distribution. Figure 19b further presents the results of the mesh-independence verification for the electro-thermo-mechanical-chemical coupled analysis. As the number of elements increases, the stress response gradually converges toward a stable value. Once the mesh reaches 4127 elements, the predicted stress exhibits negligible variation with further mesh refinement, indicating that the numerical solution is no longer sensitive to mesh density.

Accordingly, a finite element mesh comprising 4127 elements is adopted in the subsequent simulations, providing an optimal balance between computational accuracy and efficiency.

### 5.4. Finite Element Simulation of Stress and Temperature Fields

**Figure 20:**
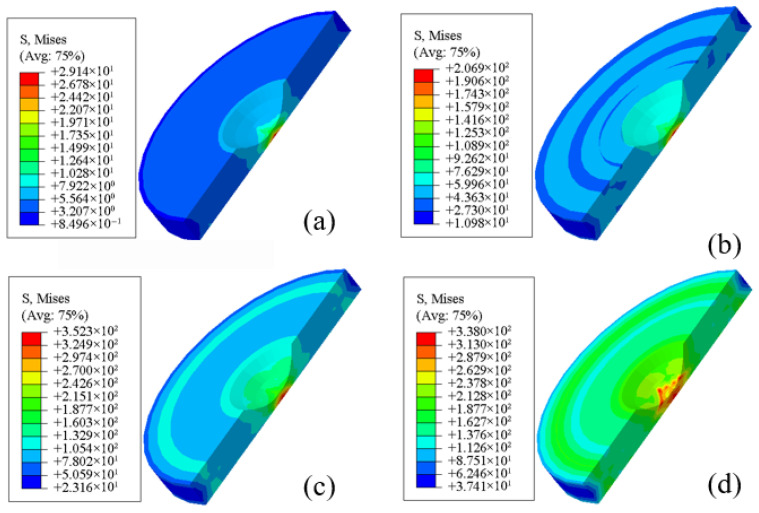
Mises stress field clouds of corrosion pits corresponding to different moments: (**a**) time = 30 s, Mises stress cloud; (**b**) time = 300 s, Mises stress cloud; (**c**) time = 600 s, Mises stress cloud; (**d**) time = 900 s, Mises stress cloud.

The finite element simulations reveal the evolution of the Von Mises equivalent stress field at the center of the corrosion pit during different stages of corrosion. Figure 20a–d present the stress contour maps at corrosion times of 30 s, 300 s, 600 s, and 900 s, respectively.

As shown in Figure 20, the equivalent Von Mises stress at the pit center exhibits a non-monotonic evolution, characterized by an initial increase followed by a gradual decrease with increasing corrosion time. At 300 s, the maximum Von Mises stress at the pit center reaches approximately 207 MPa. As the corrosion time extends to 600 s, the stress further increases to about 352 MPa. Combined with the equivalent plastic stress contours, this stress level indicates that the material in the pit region has approached or entered the plastic deformation regime under the combined action of the imposed electric current and thermo-mechanical coupling.

When the corrosion time increases to 900 s, the maximum Von Mises stress at the pit center slightly decreases to approximately 338 MPa. This reduction suggests that, under sustained electro-thermal loading, local plastic deformation and material softening progressively dominate, thereby alleviating stress concentration. Overall, the temporal evolution of stress follows a “strengthening-relaxation” trend, highlighting the significant role of thermally induced plasticity in governing the local stress field during pitting corrosion.

The stress distribution around the corrosion pit exhibits a pronounced concentric-ring pattern, with the equivalent von Mises stress at the pit center being significantly higher than that in the surrounding regions. The maximum stress is typically localized at the tips of regions subjected to strong electro-magneto-thermal coupling, reflecting stress concentration arising from the combined effects of geometric discontinuities and multiphysics interactions. These results indicate that, during corrosion, stress progressively accumulates in the pit center and decays radially outward in a concentric manner, providing important insight into the spatial distribution and temporal evolution of local stresses under corrosive conditions.

**Figure 21:**
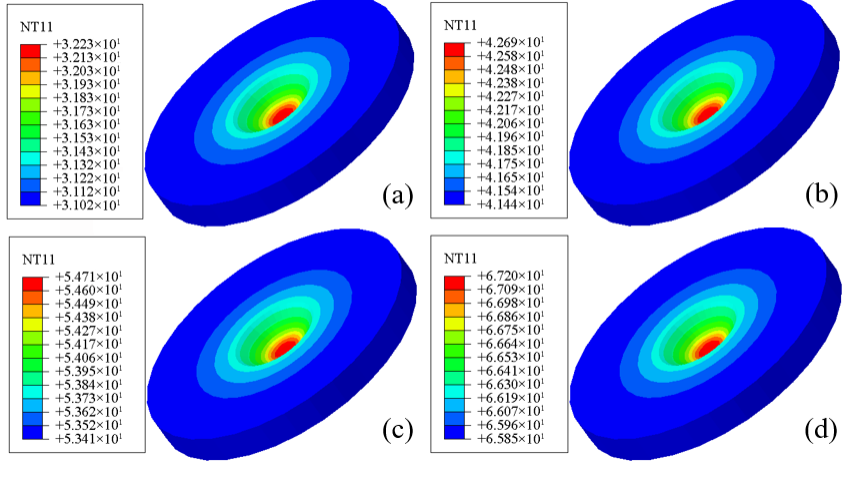
Temperature field clouds of corrosion pits corresponding to different moments: (**a**) time = 30 s, NT11 temperature cloud; (**b**) time = 300 s, NT11 temperature cloud; (**c**) time = 600 s, NT11 temperature cloud; (**d**) time = 900 s, NT11 temperature cloud.

The finite element simulations also reveal the temporal evolution of the temperature field at the pit center. Figure 21a–d show the temperature contours at corrosion times of 30 s, 300 s, 600 s, and 900 s, respectively. At the early stage of corrosion (30 s), the pit-center temperature is approximately 32 °C, close to the ambient temperature. With increasing corrosion time, the pit-center temperature rises continuously, reaching about 67 °C at 900 s. This temperature increase is primarily attributed to the accumulation of Joule heating induced by the applied electric current.

The sustained elevation of the temperature field further influences both the mechanical response and the electrochemical reaction kinetics. On the one hand, thermal softening alters the local stress distribution; on the other hand, elevated temperature may accelerate electrochemical reactions, thereby exerting a coupled effect on the growth and evolution of the corrosion pit.

The temperature distribution within the corrosion pit exhibits a pronounced concentric-ring pattern, with the temperature gradually decreasing from the pit center toward the surrounding regions and the pit center remaining significantly hotter than its periphery. This distribution indicates continuous heat accumulation within the pit, followed by radial heat dissipation into the surrounding material.

These results further confirm that, during corrosion, the pit-center temperature increases progressively over time and expands radially in a circular manner. The numerically predicted temperature field shows good agreement with the “white mist” phenomenon observed experimentally. This phenomenon can be attributed to intense Joule heating under high electric current, which elevates the local temperature to a level sufficient to trigger vaporization or gas release, thereby producing visible white fumes above the pit region.

The electro-thermo-mechanical coupled simulations successfully reproduce this thermally induced experimental feature, demonstrating the capability of the proposed model to capture localized temperature rise and its underlying physical mechanisms. This consistency provides strong support for the reliability of the model in quantitatively analyzing corrosion behavior under multiphysics coupling conditions.

### 5.5. Influence of Current on Pit Propagation Behavior

**Figure 22:**
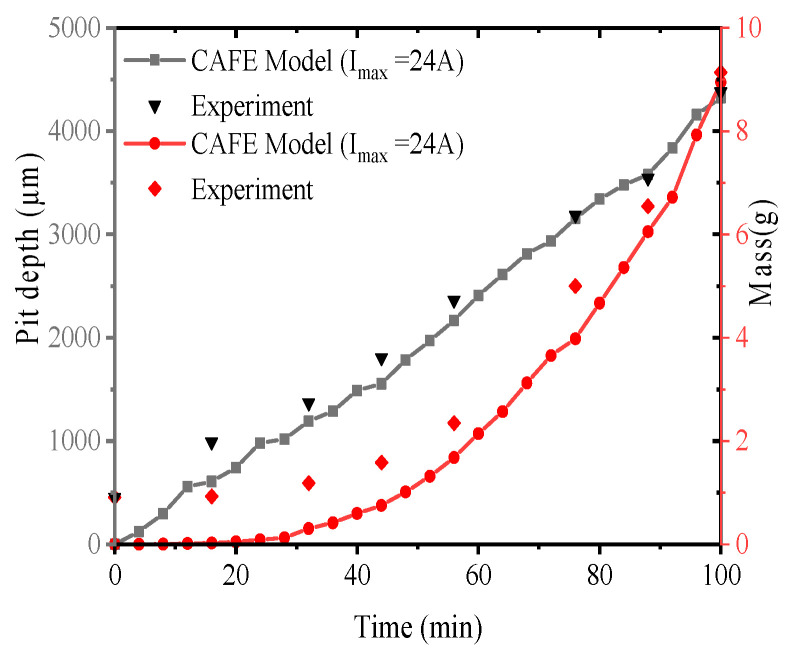
Experiment-CAFE model comparison.

To validate the accuracy and applicability of the CAFE model, a systematic comparison was conducted between the experimentally measured data and the numerical predictions obtained from the CAFE simulations, as shown in Figure 22. The results demonstrate that the model is capable of reliably reproducing the accelerated growth behavior of pitting corrosion under high-temperature, high-salinity, and high-humidity conditions. Under the application of high electric current, both pit depth and corrosion mass increase continuously with time, and their temporal evolution exhibits good agreement with the experimental observations.

Of particular interest is the observation that the time window during which stress localization exerts a pronounced influence on pit growth closely coincides with the onset of significant plastic strain development at the pit bottom. This correspondence indicates that, during the early stages of pit evolution, elastic strain in the surrounding region contributes only marginally to pit growth, whereas the initiation and progression of plastic deformation constitute the key driving factors for the accelerated expansion of the pit. This conclusion is fully consistent with the predictions of the CAFE model.

A comprehensive analysis further reveals that the substantial increase in pit growth rate under high electric current is primarily attributed to deformation of the material surrounding the pit induced by localized electrochemical effects. This deformation, in turn, promotes anodic dissolution and thereby accelerates pit propagation. Collectively, the experimental observations and numerical simulations provide converging evidence that electro-mechanical-chemical coupling plays a dominant role in governing the evolution of pitting corrosion.

## 6. Conclusions

Targeting the typical high-temperature, high-salinity, and high-humidity corrosive conditions encountered in marine environments, this study designed and developed a novel experimental setup for pitting corrosion and proposed a three-dimensional coupled Cellular Automata-Finite Element (CAFE) framework to simulate pit growth and evolution under the combined effects of elevated temperature, salinity, humidity, and high electric current. On this basis, an electro-thermo-mechanical-chemical multiphysics numerical model was established, enabling a unified description of the entire pitting damage evolution process.

The proposed approach systematically integrates three key mechanisms: (i) the core electrochemical processes governing pit growth kinetics, in which temperature and salinity are identified as critical environmental factors controlling localized corrosion rates; (ii) Joule heating-induced plastic deformation at the pit bottom under high electric current; and (iii) the coupling between electrochemical corrosion and thermally induced plastic responses. Together, these multiscale and multiphysics processes constitute the physical foundation of pitting damage evolution under aggressive environments and high-current thermo-mechanical effects.

Based on the coupled model, the temporal evolution of pit depth, depth-to-diameter ratio, and corrosion morphology was systematically evaluated under varying electrochemical parameters and applied high-current conditions. The numerical predictions show good qualitative agreement and quantitative consistency with experimental measurements, thereby validating the effectiveness and reliability of the proposed hybrid model in capturing pitting corrosion behavior under combined high-temperature, high-salinity, high-humidity, and high-current conditions.

## Figures and Tables

**Table 1:** Chemical composition of Q235B steel plates %.

C	Mn	Si	S	P	Fe
≤0.2	≤1.4	≤0.35	≤0.35	≤0.045	Other

Note: The galvanized layer thickness of the steel plates is 8 µm.

**Table 2:** States of cells in the CA model.

Location Points	Substance	Labels
Neutral solution sites	Water	W
Acidic solution sites	Hydrogen ions	H
Iron site	Metal	M
Iron(II) ion site	Reaction metal I	R
Iron(III) ion site	Reaction metal II	D
Ferrous hydroxide(II) site	Corrosion product I	P_1_
Ferric hydroxide(III) site	Corrosion product II	P_2_
Thin oxide layer	Ferric oxide	O

**Table 3:** Probability parameters used in the cellular automata model.

Parameter	Value
*P_2__Corr*	0.4
*P_Hyd1*	0.4
*P_Hyd2*	0.4
*Sed*	2
*P_diffH*	0.5
*P_diffFe* *(OH)_2_*	0.08
*P_diffFe* *(OH)_3_*	0.06

## Data Availability

The original contributions presented in this study are included in the article. Further inquiries can be directed to the corresponding author.
